# Self-Assembly of (l)‑Cysteine Molecules at Ag(110): A Scanning Tunneling Microscopy and X‑ray Photoemission Spectroscopy Study

**DOI:** 10.1021/acs.langmuir.5c04110

**Published:** 2026-03-14

**Authors:** Elina Mkrtchian, Anshika Singh, Ola Alayan, Giovanni Carraro, Marco Smerieri, Igor Píš, Silvia Nappini, Luca Vattuone, Gianangelo Bracco, Mario Rocca, Elena Magnano, Letizia Savio

**Affiliations:** † IMEM-CNR, Sede di Genova, Via Dodecaneso 33, 16146 Genova, Italy; ‡ Dipartimento di Scienze Matematiche, Fisiche e Informatiche, Università di Parma, Parco Area delle Scienze 7/A, 43124 Parma, Italy; § Dipartimento di Fisica, Università di Genova, Via Dodecaneso 33, 16146 Genova, Italy; ∥ CNR - Istituto Officina Dei Materiali (IOM), S.S. 14 km 163.5, 34149 Basovizza, Trieste, Italy; ⊥ Elettra-Sincrotrone Trieste S.C.p.A., S.S. 14 km 163.5, 34149 Basovizza, Trieste, Italy

## Abstract

Organic–inorganic interactions are at the basis of relevant applications, ranging from biocompatibility and nano­(bio)­technology to antifouling and hygiene issues. A fundamental understanding of the processes occurring at the interface between molecules of biological interest and inorganic surfaces is a key issue to optimize performance in this field. In the present paper, we report on the self-assembly and thermal evolution of the amino acid (l)-cysteine sublimated on Ag(110) under ultrahigh vacuum conditions. The morphology of the layer is investigated by scanning tunneling microscopy, and the chemistry of the system is determined by X-ray photoemission spectroscopy. The molecules adsorb on the surface at RT in the zwitterionic form, binding to the surface via dehydrogenation of the thiol group and, in part, of the carboxylate. When annealing the surface, they convert into the anionic form, and the morphology of the layer changes, indicating the presence of several local minima in the energy diagram of the Cys/Ag(110) system. Based on experimental evidence, empirical models of some self-assembled structures are proposed.

## Graphical abstract

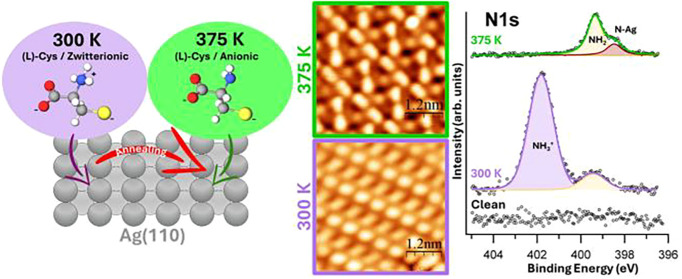

## Introduction

Adsorption of biomolecules on metal surfaces is at the basis of the understanding of interface phenomena in biomaterials, biocompatibility, and chiral recognition.^1–3^ Their self-assembly, i.e., the spontaneous arrangement of the molecules in ordered and periodic structures, is of great interest for research due to the wide range of potential applications, including biology and biomedicine,^4^ catalysis,^5^ plasmonics,^6^ and many other scientific and technological fields.^7^

Amino acids are an interesting class of molecules in this respect: they are chiral, they are the basic constituents of peptides and proteins, and they are simple enough to serve as model systems for fundamental investigation. Over the past 20 years, the adsorption of amino acids on metal surfaces has been studied using surface science techniques to unravel the adsorption modes, the two-dimensional (2D) surface organization, and the fundamental aspects of molecule–surface interactions.^8,9^

Among the 20 α-amino acids present in nature, l-cysteine (Cys) is unique since it is one of the two sulfur-containing species and the only one with a thiol group, which serves as an additional anchoring point and favors the metal–organic interaction. Therefore, Cys can be useful as a linker to graft other molecules on the surface, thus creating an interface of increased complexity,^10^ but it can also act as a promoter in the growth of noble metal nanostructures.^6^

Scientific research on the adsorption of amino acids at metal surfaces^8,9,11–16^ has mainly focused on coinage metals, since their limited reactivity should allow intermolecular interactions to prevail over molecule–substrate attraction and favor the formation of self-assembled layers, leaving the molecular structure nearly unaltered. For Cys, Au surfaces were initially privileged as substrates due to the presence of the thiol group, which ensures adsorption and eventually chiral transfer even on the least reactive metal. This justifies the significant experimental and computational effort invested in understanding this system, as well as the extensive literature on the topic.^17–24^ Indeed, Cys forms a wide variety of self-assembled geometries both on Au(111)^25,26^ and on Au(110);^27,28^ these structures range from dimers and rows to extended 2D layers and indicate the presence of several local minima in the energy landscape of the system. Alike Au, silver also has good biocompatibility properties, but it is more reactive due to its different electronic configuration.

Such reactivity, intermediate between Au and Cu, could potentially lead to the formation of stable layers while maintaining a limited distortion of the molecular structure. Fisher et al.^29^ investigated (l)-cysteine adsorption at Ag(111) by combining scanning tunneling microscopy (STM) with synchrotron-based X-ray photoelectron spectroscopy (XPS) and near-edge X-ray absorption fine structure (NEXAFS). They found that adsorption always occurs through the deprotonated thiol group, as it is expected also from density functional theory (DFT) calculations^30^ and by analogy with Au surfaces;^17–19^ the zwitterionic or anionic state of the molecule as well as the self-assembled geometry depend on surface temperature. On the Si(111)-√3 × √3-Ag surface, a similar bonding picture is observed at room temperature (RT) from combined XPS and STM data and DFT calculations.^14^ Indeed, chemisorption in the zwitterionic form and through −S–Ag bond formation is detected. Cys islands start to nucleate near the step edges, and with increasing coverage, they evolve eventually into disordered coral-reef-shaped islands on the terraces of the Si(111)-√3 × √3-Ag surface. Due to the monolayer thickness of the Ag film, Cys adsorption induces formation of Ag clusters above 175 °C. While the cited literature refers to Ag surfaces or films with (111) symmetry, much less work has been done to date on Cys interaction with the other high Miller index Ag planes. In particular, to the best of our knowledge, no systematic study has ever been performed on Ag(110), except for a limited reflection anisotropy spectroscopy study.^31^ The open atomic arrangement is expected to favor a higher reactivity than the more compact Ag(111) and Ag(100) faces.

In this study, we fill this gap by reporting a combined XPS and STM investigation of the self-assembly and thermal evolution of Cys molecules deposited on Ag(110) at room temperature. The molecules anchor to the surface via the thiolate group and, in some cases, via the carboxylate group. They self-assemble into various geometries, coexisting on the surface and changing with annealing temperature, indicating the presence of several local minima in the energy landscape of the self-assembled patterns. Adsorption of Cys molecules in the zwitterionic form is dominant on Ag(110) at RT, while by annealing the surface above 350 and 400 K, respectively, we observe conversion into the anionic state and dissociative desorption, leaving S atoms at the surface. Empirical models based on experimental data are proposed for the most common molecular arrangements.

## Experimental Section

### Sample Preparation

The Ag sample, a single crystal cut less than 0.1° off the (110) plane, was cleaned by cycles of sputtering with noble gas ions (either Ne or Ar) followed by annealing to a temperature T = 873 K or T = 773 K, respectively. (l)-cysteine (Sigma-Aldrich, > 97% purity) was sublimated at a temperature 368 K ≤ *T*_sub_ ≤ 377 K from a homemade quartz crucible mounted on both ultrahigh vacuum (UHV) apparatuses used to perform the experiments. Careful outgassing through a differential pumping line was performed before each evaporation. The Ag(110) sample was held at RT during Cys deposition. The surface was then step-annealed to 450 K to investigate the different self-assembled geometries.

### Experimental Methods

The microscopy experiments were carried out in an UHV system operating at a base pressure of ∼5 × 10^–9^ mbar and equipped with a low-temperature scanning tunneling microscope (LT-STM, manufactured by CreaTec), a homemade evaporator, and all standard vacuum facilities for sample cleaning and residual gas analysis.

STM images were recorded at liquid nitrogen temperature (LN_2_, 77 K), using a Pt–Ir tip. The images were acquired in constant current mode with a bias voltage of −2.2 V ≤ V ≤ 0.5 V applied to the sample. Atomically resolved images of the clean Ag(110) sample, as the one reported in the inset of Figure 1A, were used to determine surface orientation and to calibrate image dimensions. The images were processed using the WSxM software.^32^

**Figure 1:**
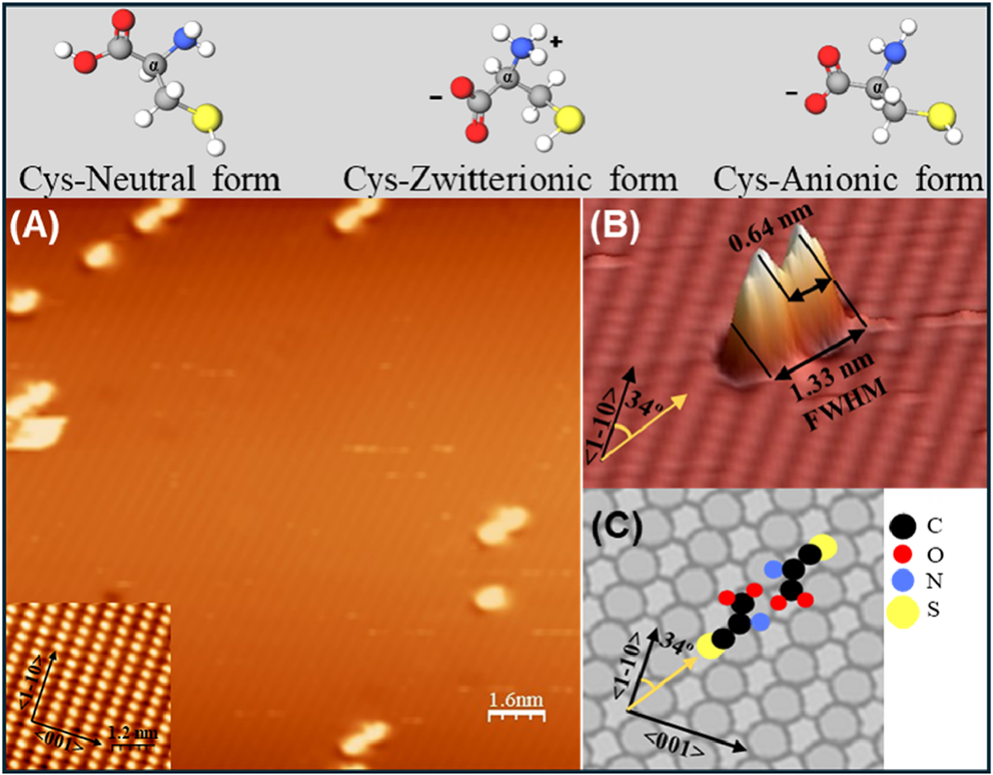
Top row: molecular structure of (l)-cysteine in the neutral, zwitterionic and anionic forms, respectively. The carbon atom corresponding to α-C (yielding the C2 contribution to the C 1s XPS spectra) is marked by α. Bottom panels: LT-STM images of (L)-cysteine molecules deposited onto Ag(110) at RT in the very low coverage limit. (A) Overview of the Ag(110) surface (16 × 16 nm^2^, *V* = −0.42 V, *I* = 1.8–10 A), showing a few isolated ad-molecules and dimers on the surface. The inset shows an atomically resolved image of the clean Ag(110) surface (4 × 4 nm^2^; V = −2.1 V), used for calibration. The high symmetry directions are marked by arrows. (B) 3D view of a Cys dimer. (C) Empirical model deduced from STM images and XPS information. Here and in the following models, hydrogen atoms are not reported for easier visualization.

The composition and chemical state of the Cys layers were analyzed by High-Resolution X-ray Photoemission Spectroscopy (HR-XPS) at the BACH (Beamline for Advanced di CHroism) beamline of the Elettra synchrotron radiation source in Trieste (Italy).^33^ HR-XPS spectra were acquired using linearly polarized light with photon energy *hν* = 372 eV for S 2p and C 1s regions, *hν* = 522 eV for N 1s and Ag 3d regions, and *hν* = 596 eV for the O 1s region. The total instrumental resolution was 0.2 eV. The binding energy (*E*_b_) was calibrated on the Fermi Edge; this procedure yields an Ag 3d_5/2_ line at 368.2 eV, in good agreement with literature values.^34^ In each region, spectra were normalized on the low *E*_b_ side of the clean surface spectrum to allow for an easier comparison. They were fitted using KolXPD software after background subtraction. A Shirley background was employed for the C 1s and S 2p regions, while a linear background was applied to the N 1s and O 1s ones. Voigt functions were used to fit all regions except the S 2p region, for which a Voigt doublet was employed.

## Results and Discussion

Figure 1A shows a typical STM image obtained after sublimation (*T*_sub_ = 368 K) of Cys for 10 min on the Ag(110) surface held at RT. As evident, only a few isolated features are present on the surface, indicating that a very low Cys coverage has been achieved. These features are either roundish or 8-shaped. Their dimensions are compatible with isolated Cys molecules and with Cys dimers, respectively (see Figure 1B for an enlarged three-dimensional (3D) view of the dimer). The formation of dimers at such low coverage indicates a strong tendency of the molecules to diffuse on the surface and aggregate.

After 20 min of deposition on the Ag(110) sample at RT and higher flux (*T*_sub_ = 377 K), the Cys layer appears significantly different (see Figure 2A,B): now the ad-molecules are arranged in a regular, periodic pattern that fully covers the flat terraces of the surface, while a different, less ordered arrangement is observed in stepped areas. As evident from the high-resolution image in Figure 2A, the self-assembled structure consists of rows of brighter round features alternating with lower, elongated ones. A rhomboidal unit cell can be identified by vectors *a⃗* (*a* = 1.08 nm), along the <1–10> direction, and *b⃗* (*b* = 0.56 nm) along the rows and −80° off <1–10> (see line scans in Figure 2C and Table 1 for summary of unit cell parameters). Each unit cell contains one round and one oval feature (referred to as unit 1and unit 2 in the following); the former has a diameter of ∼0.35 nm (fwhm), while the latter has the same width but it is ∼0.7 nm long, with its axis oriented 34° off <1–10> (similar to the orientation of isolated dimers in Figure 1). They are therefore likely to correspond to two Cys units adsorbed on the surface in a different configuration. Considering the size of the unit cell, we can estimate a surface density of ∼3.3 × 10^14^ molecules/cm^2^, corresponding to 0.39 ML (in monolayers of Ag(110)).

**Figure 2:**
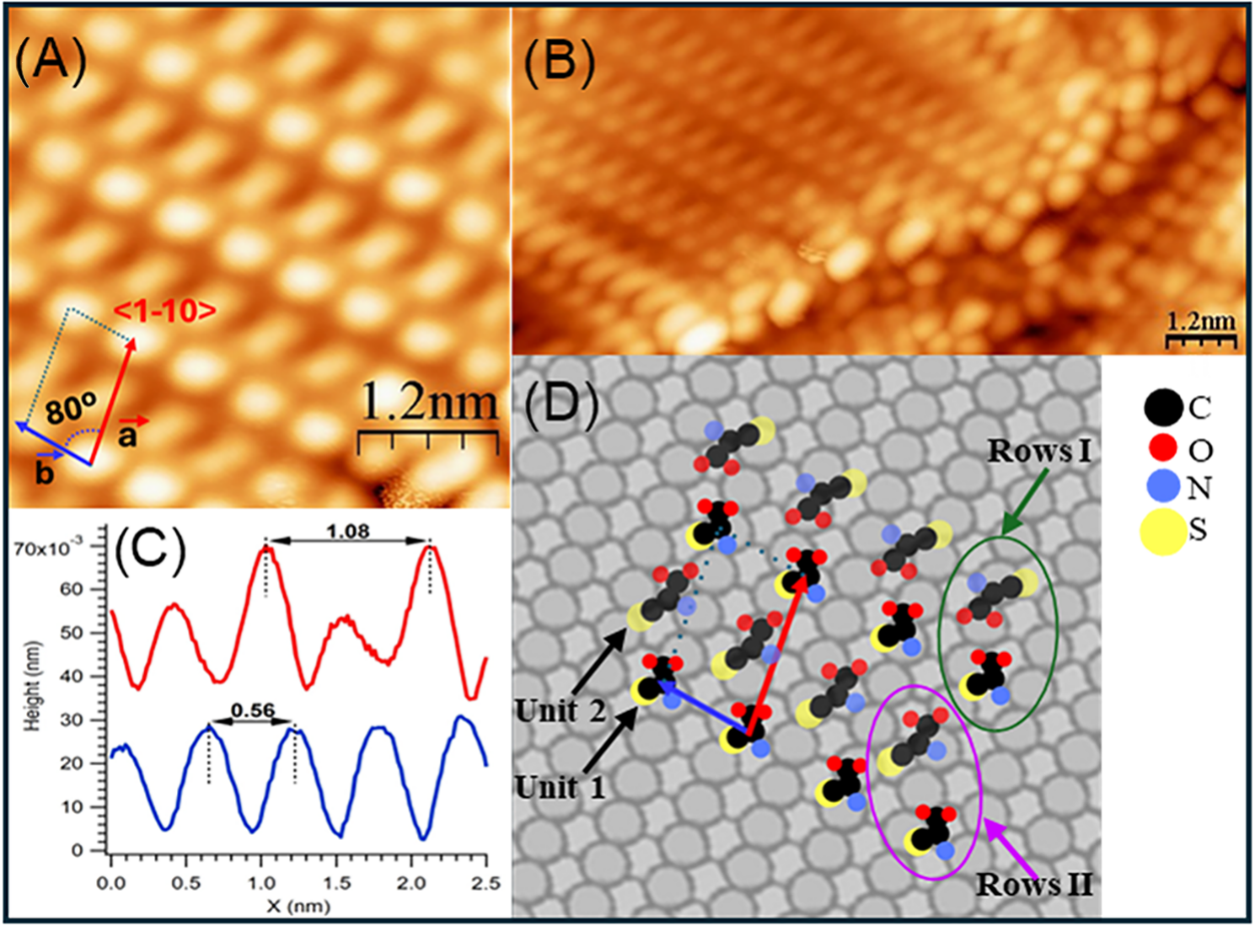
LT-STM images of the (l)-cysteine self-assembled layer observed after deposition onto the Ag(110) surface at RT: (A) High-resolution image of the Cys rows. The unit cell (defined by *a⃗* and *b⃗* vectors and by the angle α = 80° between them) is marked. Image size: 4 × 4 nm^2^; *V* = −2 V, *I* = 2.0^–10^ A. (B) Overview of the Cys layer, showing the ordered assembly at the terrace and a disordered arrangement close to steps. Image size: 12.1 × 5.8 nm^2^, *V* = −2 V, *I* = 2.0^–10^ A. (C) Line scans cut along the unit cell directions, from which the size of the unit cell is determined. (D) Empirical model deduced from STM images and from XPS information. Different possible orientations of unit 2 are sketched in rows I and II, respectively. The relative position of the different functional groups is tentative.

**Table 1:** Characteristic Parameters of the Unit Cell for the Different Self-Assemblies Shown in Figures 2 and 4 ^a^

figure	T (K)	length of the vectors *a⃗* and *b⃗* (nm)		angle γ (°)	angle α (°)
Figure 2A	300 (RT)	1.08	0.56	0	80
Figure 4D	325	0.51	1.14	11	95
Figure 4E	375	2.60	1.20	33	70

a The *a⃗* vector is oriented off the <1–10> direction by the γ angle; the *b⃗* vector is rotated counterclockwise with respect to *a⃗* by the α angle.

The HR-XPS spectra corresponding to a (l)-cysteine/Ag­(110) layer similar to that in Figure 2 are reported in Figure 3 (bottom traces). Indeed, deposition at RT was performed under the same experimental conditions, except for the deposition time, which was increased to 40 min to compensate for the larger crucible-surface distance in the experimental chamber at the BACH beamline. The S 2p, C 1s, N 1s, and O 1s regions are shown in Figure 3 panels A–D, respectively, and the position and assignment of the main features are summarized in Table 2. In each region, the spectrum of the clean surface is also reported for reference. The S 2p region is characterized by a single doublet with *E*_b_(S 2p_3/2_) = 161.8 eV (S1) and spin–orbit separation Δ*E* = 1.2 eV; it is ascribed to deprotonated sulfur bound to the silver surface, in perfect agreement with what was previously reported for Cys/Ag(111) and, more in general, for thiol groups at noble metals.^29,35^

**Figure 3:**
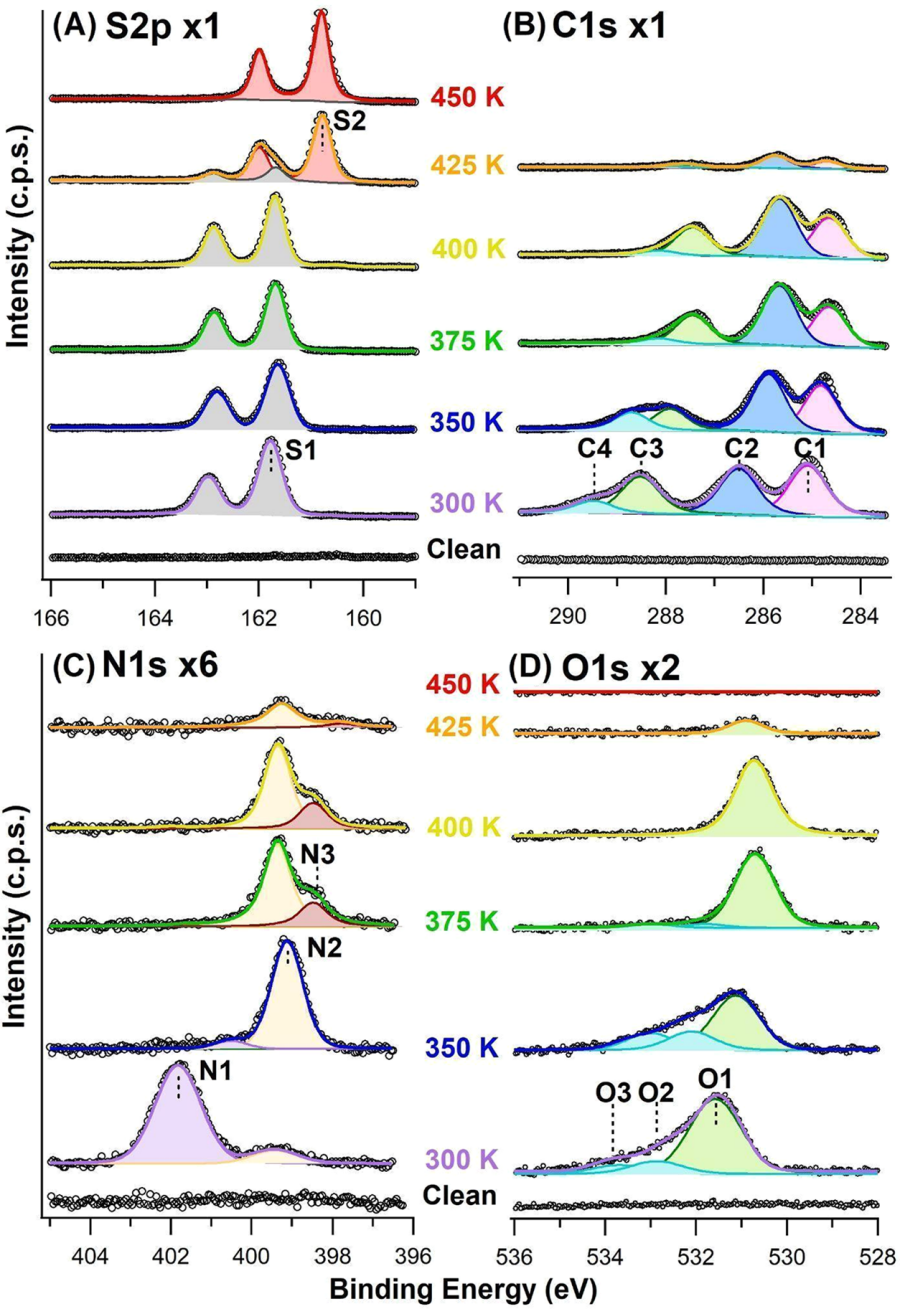
HR-XPS spectra of the (l)-cysteine/Ag­(110) layer after 40 min of deposition at RT and subsequent step annealing to the indicated temperature. (A) S 2p region; (B) C 1s; (C) N 1s; (D) O 1s. All spectra are acquired at RT; spectra of the clean surface are reported as reference. The different regions are plotted using the same intensity range, so that details can be better appreciated, including those in the spectra of the lowest intensity. Differences in the intensity of the corresponding S 2p, C 1s, N 1s and O 1s lines, due to the different amounts of those elements and to the photoionization cross sections of the specific line, can be appreciated considering the magnification factor marked in each panel.

**Table 2:** XPS Core Level Peak Assignments of the S 2p, C 1s, N 1s, and O 1s Lines Determined from the Fitting of the Spectra of Figure 3, Corresponding to a (l)-Cysteine Layer Deposited on Ag(110) at RT and Subsequent Annealing Steps^a^

*E*_b_(eV)	label	assignment
160.8	S2	Ag–S–Ag^37^
161.6–161.8	S1	–C–S-Ag^29,38^
284.6–285.1	C1	C–S^9,14,29^
285.7–286.5	C2	α-C^9,14,29^
287.4–288.5	C3	COO^-^ ^9,14,29^
288.2–289.5	C4	COOH^9,14,29^
398.5	N3	N–Ag^39,40^
399.3–399.4	N2	NH_2_ ^9,29^
401.8	N1	NH_3_^+^ ^9,29^
530.7–531.5	O1	COO^-^ ^18,29^
531.9–532.6	O2	COOH^18,29^
533.3–533.8	O3	COOH^18,29^

a Assignments are based on comparison with the cited works on other Ag faces or on other coinage metal surfaces

The C 1s region shows three peaks of comparable intensity at *E*_b_ = 285.1 eV (C1), 286.5 eV (C2), and 288.5 eV (C3) and a minor one at 289.5 eV (C4). Considering the structure of the molecule and by analogy with previous experiments on Ag(111)^29^ and Si(111)-√3 × √3-Ag,^14^ these components can be identified, respectively, with C atoms bound to deprotonated S, with the α-C bound to the amino acid groups, and with C atoms in carboxylate and in carboxylic groups.^29^ The good correspondence with the 1:1:1 intensity ratio expected for the three inequivalent C atoms of the Cys molecule (see Table 3 for the ratio between C1, C2, and C3 + C4 lines) supports this assignment, which is further confirmed by the analysis of the N 1s (see Figure 3C) and O 1s (Figure 3D) spectra. The N 1s region is characterized by a prominent peak centered at *E*_b_ = 401.8 eV (N1) that is characteristic of the positively charged ammonium group NH_3_+ ^9,29^ and proves that Cys molecules adsorb on Ag(110) in the zwitterionic form. This is not in contrast with the presence of a fraction of undissociated carboxylic groups, since the formation of unconventional zwitterions through proton transfer from the thiol group was observed both for Cys multilayers on TiO_2_ ^36^ and for submonolayer Cys/Au(110).^18^ Indeed, this was calculated to be the most stable conformation for isolated Cys/Au(111).^21^ The minor intensity at *E*_b_ = 399.4 eV (N2), corresponding to amino acids in the anionic form,^9^ is likely to be due, at least in large part, to a photon-induced effect. In fact, we demonstrated that the intensity of this line could be reduced by minimizing exposure to the photons during uptake experiments (see Figure S3 of the Supporting Information), i.e., by recording each new spectrum on a fresh spot. The O 1s spectrum can be reproduced by a major component at *E*_b_ = 531.5 eV (O1) and by two minor components at 532.6 eV (O2) and 533.8 eV (O3). By comparison with the literature,^14,18,29^ the O1 peak is attributed to oxygen atoms in deprotonated carboxylate groups (—COO^–^), while the O2 and O3 species correspond to the two inequivalent oxygen atoms of the carboxylic groups (—COOH). The semiquantitative analysis of the C 1s and O 1s components reported in Table 3 supports this interpretation of the data, showing that the intensity ratio between C3 and C4 (C3/C4 ∼ 3.1) is in good agreement with that of the corresponding oxygen species (O1/(O2 + O3)∼3.5).

Considering the chemical information provided by XPS and the geometry of the self-assembled layer observed by STM, we can construct the empirical model shown in Figure 2D for the arrangement of the two Cys molecules within the unit cell. Unit 1 appears round and brighter in STM images; therefore, it is reasonable that it is in an upstanding configuration. Unit 2 is of elongated shape and lower apparent height, and it is likely to lie on the surface. Since all thiol groups are deprotonated, both molecules must bind to the substrate through an —S—Ag bond. For unit 1, this determines the orientation with the thiolate at the surface and the amino acid termination in the form of unconventional zwitterion (R–CH– NH_3_^+^COOH) protruding toward the vacuum. Unit 2, on the contrary, is a Cys zwitterion (R–CH– NH_3_^+^COO^–^); from comparison with different amino acid systems at coinage metals,^9^ in this case, the deprotonated carboxylate group is likely to act as a second anchoring point in bidentate configuration while the amino groups are most probably pointing upward. Finally, Figure 2D reports two possible configurations in which unit 2 points toward the other Cys molecule in the same unit cell with the amino acid termination (row I) or with the sulfur termination (row II), respectively. It is also reasonable that hydrogen bonds form between adjacent molecules, but our empirical model cannot reach this level of detail. This picture is consistent with the broader shape of the N1 peak in Figure 3C with respect to the N2 and N3 ones, since it arises from the superposition of photoemission lines from N atoms in a slightly different chemical environment (possibly related to conventional and unconventional zwitterions, respectively). We also mention that the excess intensity of the C3 component with respect to C4 is justified considering that the XPS signal comes from a macroscopic area, so that it contains both the contribution of Cys molecules in the ordered self-assembly of Figure 2A and of Cys units disorderly adsorbed close to steps and defects (Figure 2B). Since these low-coordinated sites are more reactive, molecules are likely to deprotonate and adsorb there also via the carboxylate group.

The thermal evolution of the layer up to complete molecular dissociation is addressed in the upper spectra of Figure 3 and in the parallel STM experiment reported in Figure 4.

Measurable chemical shifts in the XPS spectra are observed in every region upon annealing, indicating that the Cys molecules undergo relevant chemical transformations and, reasonably, that the self-assembled geometry also changes upon heating. Meanwhile, some reduction in the peak intensity, indicative of limited molecular desorption/dissociation, is observed; this is addressed in Figure 6, while Table 3 summarizes the changes in the relative amount of the different C 1s and O 1s components. When annealing from RT to 350 K, the C1–C4 components shift to 284.8, 285.8, 287.8, and 288.6 eV, respectively, and their relative intensities change slightly. In particular, C4, corresponding to C atoms in the —COOH groups, shows the largest downshift (Δ*E*_b_ = −0.9 eV) at 350 K and it almost disappears at 375 K, suggesting full deprotonation of the acidic groups. Such an interpretation is supported by inspection of the O 1s and N 1s regions. Indeed, only the O1 component is present after annealing to T = 375 K, while the main N 1s peak at *E*_b_ = 401.8 eV narrows and shifts to *E*_b_ = 399.1 eV already after the first annealing to 350 K. Such a shift is indicative of a chemical modification of the adsorbate, passing from a zwitterionic to an anionic state. An additional minor component (N3) appears at *E*_b_(N 1s) = 398.5 eV at 375 K and at 400 K. We tentatively assign it to N atoms subject to a larger charge transfer from the Ag substrate, in analogy with the interpretation accepted for dimethylformamide at colloidal Ag nanoparticles,^39^ for which the same E_b_(N 1s) was detected as major component. Its presence is therefore indicative that a minority of molecules are adsorbed in a different configuration, possibly at defective/disordered sites.

**Table 3:** Relative Intensity of Carbon and Oxygen Components after Deposition of (l)-Cysteine on Ag(110) at RT and upon Annealing up to 400 K^a^

	relative intensity/%			
assignment	300 K	350 K	375 K	400 K
C1	35	33	30	31
C2	32	40	45	44
C3	25	15	21	21
C4	8	12	4	4
O1	78	61	91	95
O2	13	23	5	3
O3	9	16	4	2

a Please note that the total intensity is not the same for all temperatures. The error on each component is approximately ± 5%

Finally, we observe that the behavior of sulfur is very different from the other elements since the S 2p doublet with the main peak at *E*_b_ = 161.8 eV (S1) remains almost unaltered up to 400 K, except for a small downshift of 0.1–0.2 eV. Above this temperature, it converts into a doublet of similar intensity with the main peak at *E*_b_ = 160.8 eV (S2), corresponding to bridge Ag—S—Ag bonds,^37^ while the intensity of the corresponding C 1s, O 1s, and N 1s lines decreases dramatically (see top spectra in Figures 3 and 6). The overall information indicates then that thermal decomposition of the molecule occurs above 400 K,^29,38^ during which the organic fragments desorb, leaving the S atoms at the surface.

The different panels of Figure 4 show the morphological changes of the Cys layer of Figure 2 upon step annealing to 325, 375, and 400 K, respectively. For 325 and 375 K, the overview is presented together with a high-resolution image of the dominant self-assembled structure and with the line scans from which the unit cell parameters are determined.

At 325 K, the self-assembled layer is very similar to that already reported at RT (see the overview in Figure 4A and the enlarged image in panel D). The row structure is preserved, but the overview shows that the orientations of the Cys rows on different terraces are not always the same, indicating a flexible registry with the substrate. The unit cell becomes slightly rhombohedral with respect to the one identified at RT (see line scans in Figure 4G and Table 1), suggesting that some relaxation of the layer has begun. This behavior is consistent with the XPS data, which show only a slight reduction in intensity upon annealing from 300 to 350 K, and it is not in contrast with the observed transition from the zwitterionic to the anionic form of the adsorbate. Annealing to 375 K results in a significant change in the morphology of the self-assembly: large areas of the surface are covered by a Cys layer formed by rows running 70° off <1–10> (see Figure 4B, 4E). They consist of bright and slightly elongated lobes that, considering the periodicity of 0.6 nm measured in the line scans (Figure 4H), correspond most reasonably to single Cys units. Adjacent rows are 1.4 nm apart and are connected by dimers aligned 33° off <1–10>. These dimers have a separation between maxima of 0.6 nm, compatible with Cys dimers as the one in Figure 1 and with those previously reported for Cys/Au(111).^26^ The unit cell contains two units from the rows and one dimer; hence, a total of four Cys molecules. Therefore, we can estimate a surface density of ∼2.4 × 10^14^ molecules/cm^2^, i.e., of 0.28 ML. The presence of a less dense overlayer, compared to that observed at RT, is consistent with partial desorption of the adsorbate upon annealing. Additional fainter lobes present within the unit cell can be ascribed to Ag atoms due to additional surface disordering of the substrate.^41–45^ The mechanism may be similar to the one observed on the same surface for low-temperature adatom extraction in the presence of oxygen.^46^ The same structure holds at 400 K (see Figure 4C and Table 1 for the unit cell parameters).

Since XPS indicates that anionic states dominate at T = 375 K (see Figure 3), with thiol and carboxylic groups being fully deprotonated, we propose the empirical model shown in panel I for the observed geometry. Rows are formed by chemisorbed Cys units in the anionic form, anchored at the surface with both the thiol and the carboxylate groups, while the neutral amino group might be in a lateral position or protruding toward the vacuum. Since all of the corresponding features in STM images are equivalent, we suggest that all of the Cys units have the same molecular orientation, with their axis approximately parallel to the row direction. These rows are connected by dimers of Cys molecules in the same deprotonated form. The Cys units of the dimers appear as two triangles pointing toward each other. Since this shape is peculiar to the dimer units and not Cys molecules in the rows, we rule out a tip effect and assume that the molecules in the dimer are rotated by 180° with respect to each other. At this stage, we cannot determine whether they are facing with the sulfur or carboxylate terminations; however, we can certainly exclude intermolecular interaction through formation of a S–S bond, which is expected to give an XPS signal above 163 eV,^40,47^ contrary to our experimental evidence. A theoretical DFT model would be necessary to better understand the conformation of Cys on the Ag(110) surface at a greater level of detail.

The overviews in Figure 4B,C show that different molecular arrangements are also present on the surface as minority structures. In particular, at 375 K, the more complex assembly reported in the enlarged image of panel F is present. It consists of a dense assembly of round features, from which brighter clusters formed by eight lobes emerge. These clusters have an almost square shape with the sides aligned along the high symmetry direction of Ag(110) and a distance between the external maxima of approximately 1 nm. The single bright spots are therefore likely to be Cys units in a compact arrangement, surrounded by other molecules or by Ag atoms displaced from the surface. We notice that a similar arrangement was previously observed for Cys/Au(110) – (1 × 2).^48^ In that case, however, the Cys clusters were isolated on an ordered surface, which can explain the larger dimensions reported. This assembly is not observed upon heating to 400 K, where the minority structure (visible in the bottom-left corner of panel C) has a striped appearance.

**Figure 4:**
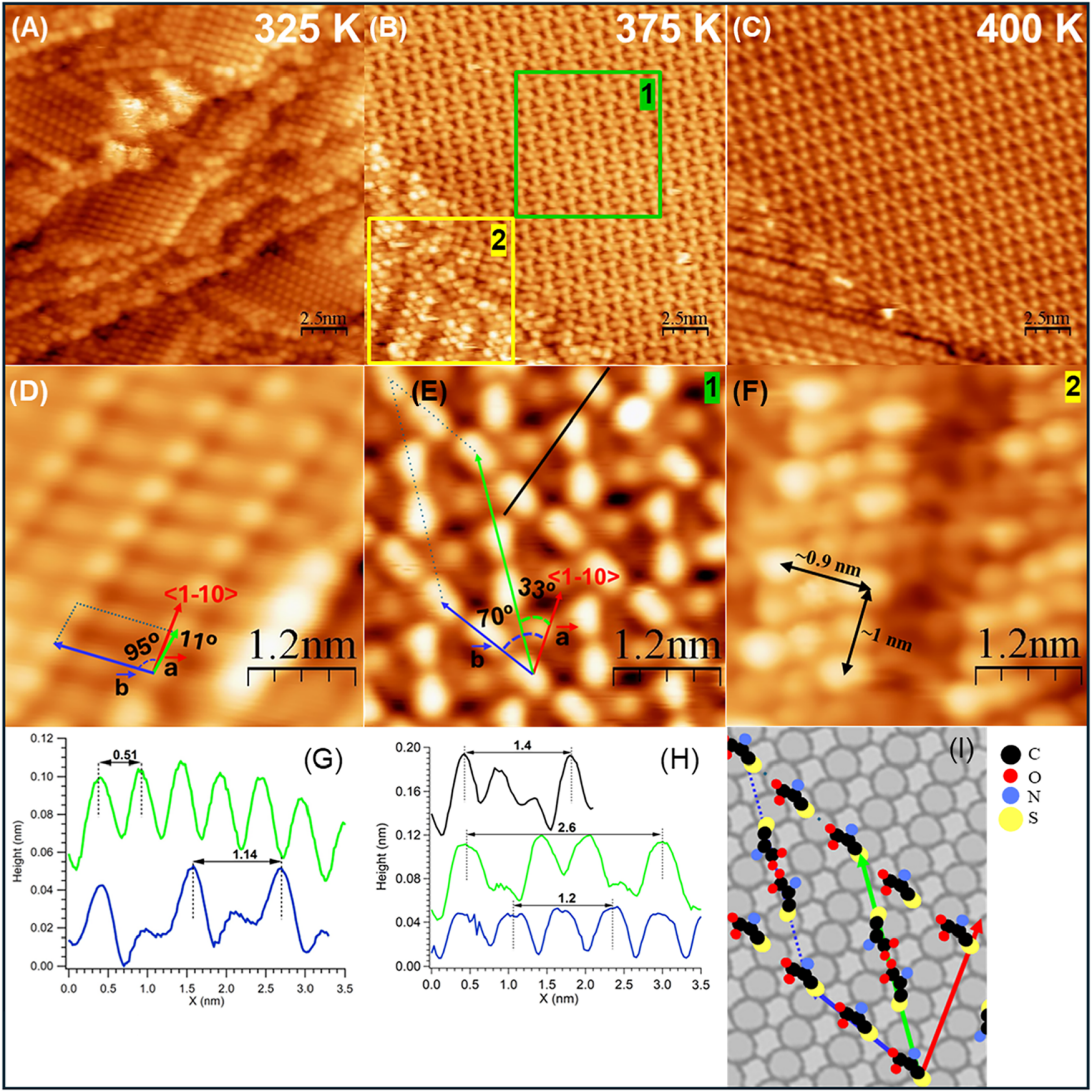
LT-STM images of self-assembled Cys obtained after annealing the layer. Top row: overview of the Cys self-assembled observed after annealing to 325 K ((A), *V* = −2.2 V), 375 K ((B), *V* = −1.5 V), and 400 K ((C), *V* = −2.2 V). For all images, size: 20 × 20 nm^2^. Central row: high-resolution images of the Cys structures observed at 325 K ((D), *V* = −2.2 V) and at 375 K ((E, F), *V* = −1.5 V and −2.0 V, respectively). The unit cell (defined by *a⃗* and *b⃗* vectors and by the α angle between them) is marked in panels D and E. For all images, size: 4 × 4 nm^2^. Bottom row: panels G/H report the line scans cut along the unit cell directions in panels D/E, respectively, from which the size of the corresponding unit cells can be determined. Panel I shows the empirical model of the structure in E, as derived from STM images and XPS spectra.

Annealing above 400 K leads to the formation of the nonperiodic geometries reported in Figure 5: the surface is covered by single and double features. The latter are “butterfly-shaped” and always present the same orientation, that is, with the long axis approximately 60° off <1–10>. The number of dark holes in between them increases significantly from 425 to 450 K (compare panels A and B of Figure 5). Since XPS data indicate that almost complete desorption of Cys occurs above 400 K and that only S atoms remain at the surface, we propose that the bright features in the assemblies of Figure 5 correspond to individual S atoms bound to Ag at different adsorption sites. This interpretation is reinforced by considering that the density of such features is ∼0.32 ML, which is in good agreement with the 0.28 ML coverage of Cys molecules in the 375 K assembly.

We note that S binds readily to Ag, possibly leading to the formation of overlayers and surface reconstruction.^42,43^ Preferential adsorption at bridge sites has been predicted by DFT calculations on methyl-thiolate at Ag(111),^42^ while also the formation of AgS_2_ complexes is reported for Ag(110) exposed to S_2_ (gas) at RT.^45^ Our experimental conditions are not comparable with those of,^45^ since in our case the S adlayer is due to dissociation of an organic molecule, and we have no evidence of the formation of AgS_2_. However, we notice that the butterflies tend to align their long axes parallel to one another and form rows nearly parallel to the step edge. Hence, based on the cited literature and on the ∼0.4 nm distance between the maxima (see line scan in Figure 5D), we propose that isolated features correspond to single S atoms and that the butterflies consist of two S atoms in next-nearest-neighbor sites, giving a configuration that resembles the zigzag one given in ref. 45

**Figure 5:**
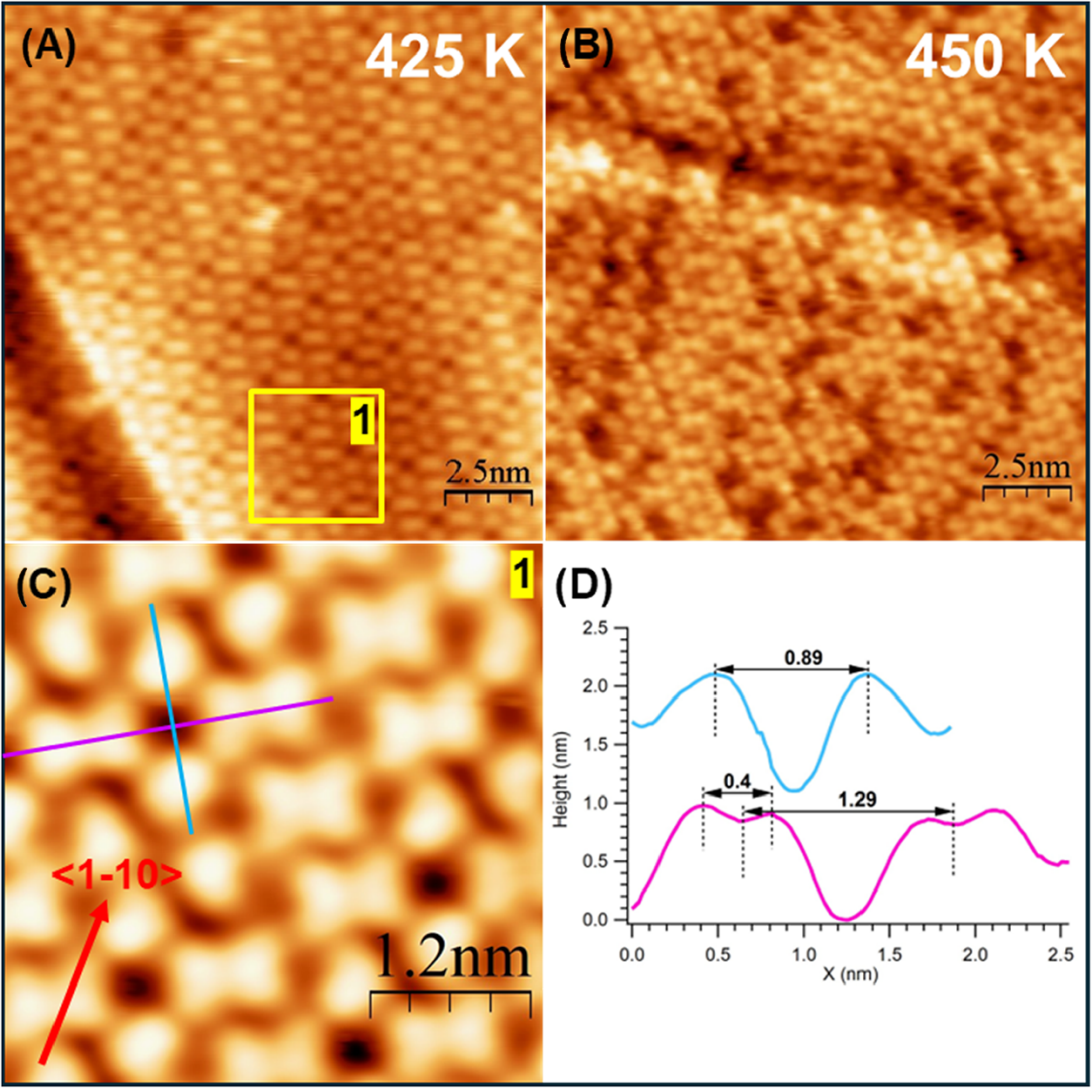
LT-STM images of the Cys/Ag(110) surface after annealing the layer to 425 K ((A), *V* = −1.7 V) and 450 K ((B), *V* = −0.5 V). For both images: size 15.1 × 15.1 nm^2^. (C) High-resolution image of the area marked by the yellow box in panel A. Image size: 4 x 4 nm^2^, *V* = −1.5 V. (D) Line scans cut along the lines marked in panel C, from which the average dimensions of the different features can be determined.

Combined spectroscopic and morphological investigations of the Cys/Ag(110) system allow us to deduce the following information:

(i) When the Ag(110) substrate is at RT, Cys ad-molecules are highly mobile and have a strong tendency to self-assemble at the surface, forming monolayer structures that entirely cover the Ag(110) terraces.
(ii) Different structures form, sometimes also coexisting on the surface, depending on the annealing temperature. This is indicative that the adsorption energy landscape must have several local minima, possibly very close to each other. Cys ad-molecules organize by self-assembly, reaching structures allowed by their thermal energy. When additional energy is provided to the system by annealing, they overcome a barrier and rearrange on the surface, reaching a deeper local minimum. This behavior is common for other amino acids at Ag and Cu^9,15,16^ and has already been reported for Cys/Au(111).^26^
(iii) Cys molecules chemisorb at Ag(110) already at RT. Indeed, the thiol group is always deprotonated, indicating that it serves as the first anchoring point, regardless of the molecular configuration. When also the carboxylic group is deprotonated, a second anchoring point is available. By analogy with the extensive literature in the field,^8,9,16,25,26,29^ it is reasonable that the carboxylate binds to the surface with both oxygens, i.e., in a bidentate configuration, while the amino group points away from the surface.
(iv) Adsorbed Cys molecules are in the zwitterionic form on the Ag surface at RT and convert into the anionic form as soon as the temperature is increased, or in the presence of significant photon irradiation (see Figure S3 in SI), indicating that this is not the most stable adsorption state but only a local energy minimum.
(v) Finally, Figure 6 shows the total area (panel A) and the relative concentration (panel B) of the Cys constituents as a function of annealing temperature (as calculated from the XPS spectra of Figure 3). It allows for additional considerations regarding both the stoichiometry and the thermal stability of the Cys molecules. First, we note that the O:N:S ratio in Cys molecules adsorbed onto the Ag(110) sample at RT is 1.9:1.2:1.0, in good agreement with the expected values. The C signal is excluded because its concentration is slightly more abundant than expected. This is reasonably due to some residual carbon contamination from the crucible, as discussed in the SI. Moreover, since photoemitted electrons have kinetic energies between 80 and 220 eV, corresponding to wavelengths between 0.13 and 0.08 nm, photoelectron diffraction effects may also affect the relative intensities of the different components even if they belong to the same unit. The concentration of the Cys constituents remains nearly constant upon annealing up to 400 K, while the total area of all elements decreases, indicating partial desorption of the molecules off the Ag(110) surface. This is consistent with the observation of a less dense self-assembled geometry by STM at 375 K. Above 400 K, the intensity of the C 1s, O 1s, and N 1s lines drops abruptly; vice versa, the S 2p signal remains constant over the whole temperature range. This is reflected in the relative concentration of the different elements on the surface, which now presents a significant abundance of S. The overall information confirms that chemisorbed Cys desorbs almost entirely above 400 K; desorption occurs by breaking off the C—S bond and leaving the S atom, arranged at the surface according to the single and butterfly-shaped features of Figure 5. Such behavior is not surprising given the strong affinity of S for Ag atoms and the previously observed dissociative desorption of Cys on Au(110).^28^

**Figure 6:**
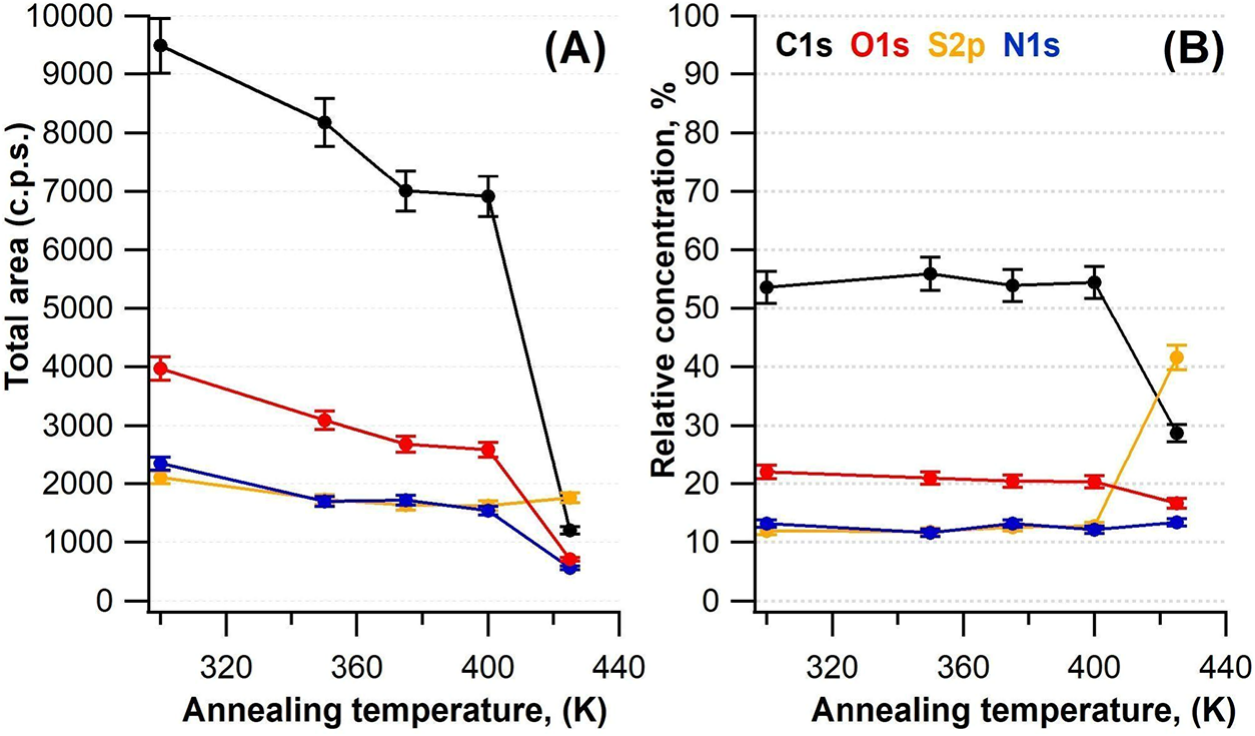
Total area (A) and the relative concentration of elements (B) versus annealing temperature. All data points are affected by an estimated error of ± 5%. The intensities and relative concentrations of all elements are scaled on the corresponding photoionization cross sections for direct comparison.^49^

## Conclusion

The self-assembly and thermal evolution of a l-cysteine monolayer produced by sublimation on Ag(110) at RT were investigated by combining LT-STM and XPS to unravel both the morphology and the chemistry of the system. At RT, Cys ad-molecules chemisorb in the zwitterionic form and form a self-assembled monolayer via a strong —S—Ag interaction, as well as the possible binding of carboxylate to the surface through both oxygens. Annealing to 350 K leads to conversion of the ad-molecules from zwitterionic to anionic and some relaxation of the adlayer. The geometry changes dramatically at 375 K, while desorption starts above this temperature and is driven by the breaking of the C–S bonds and sulfur poisoning of the surface. Our results present similarities with what was previously observed for Cys/Ag(111)^29^ and also peculiar self-assemblies related to the geometry of the substrate. We believe this fundamental characterization of the system is relevant in view of the peculiar characteristics of Cys, which is the only natural amino acid with a thiol group and that can be used as a linker for further surface functionalization.

## Supplementary Material



## Notes

HR-XPS spectra of (l)-Cysteine/Ag­(110) uptake; elemental concentration analysis; effect of photon irradiation (PDF)
