# Supplementary material for: Self-Assembly of (l)‑Cysteine Molecules at Ag(110): A Scanning Tunneling Microscopy and X‑ray Photoemission Spectroscopy Study
Source: Langmuir. 2026 Mar 14;42(12):8290–300. doi: 10.1021/acs.langmuir.5c04110 (PMC13045017; doi:10.1021/acs.langmuir.5c04110)
Supplement: Supplementary file 1 [file la5c04110_si_001.pdf]

Self-assembly of (L)-cysteine molecules at Ag(110): a scanning tunneling microscopy and X-ray photoemission spectroscopy study.

*Elina Mkrtchian,[1, 2], Anshika Singh[3], Ola Alayan[1,3], Giovanni Carraro[1], Marco Smerieri[1], Igor Píš,[4,5], Silvia Nappini[4], Luca Vattuone[1,3], Gianangelo Bracco[1,3], Mario Rocca[1,3], Elena Magnano[4], Letizia Savio[1]**

1 IMEM-CNR, Sede di Genova, Via Dodecaneso 33, 16146 Genova, Italy

2 Dipartimento di Scienze Matematiche, Fisiche e Informatiche, Università di Parma, Parco Area delle Scienze 7/A, 43124 Parma, Italy

3 Dipartimento di Fisica, Università di Genova, Via Dodecaneso 33, 16146 Genova, Italy

4 CNR - Istituto Officina dei Materiali (IOM), S.S. 14 km 163.5, 34149 Basovizza, Trieste, Italy

5 Elettra-Sincrotrone Trieste S.C.p.A., S.S. 14 km 163.5, 34149 Basovizza, Trieste, Italy

*Corresponding author: letizia.savio@cnr.it

SUPPORTING INFORMATION

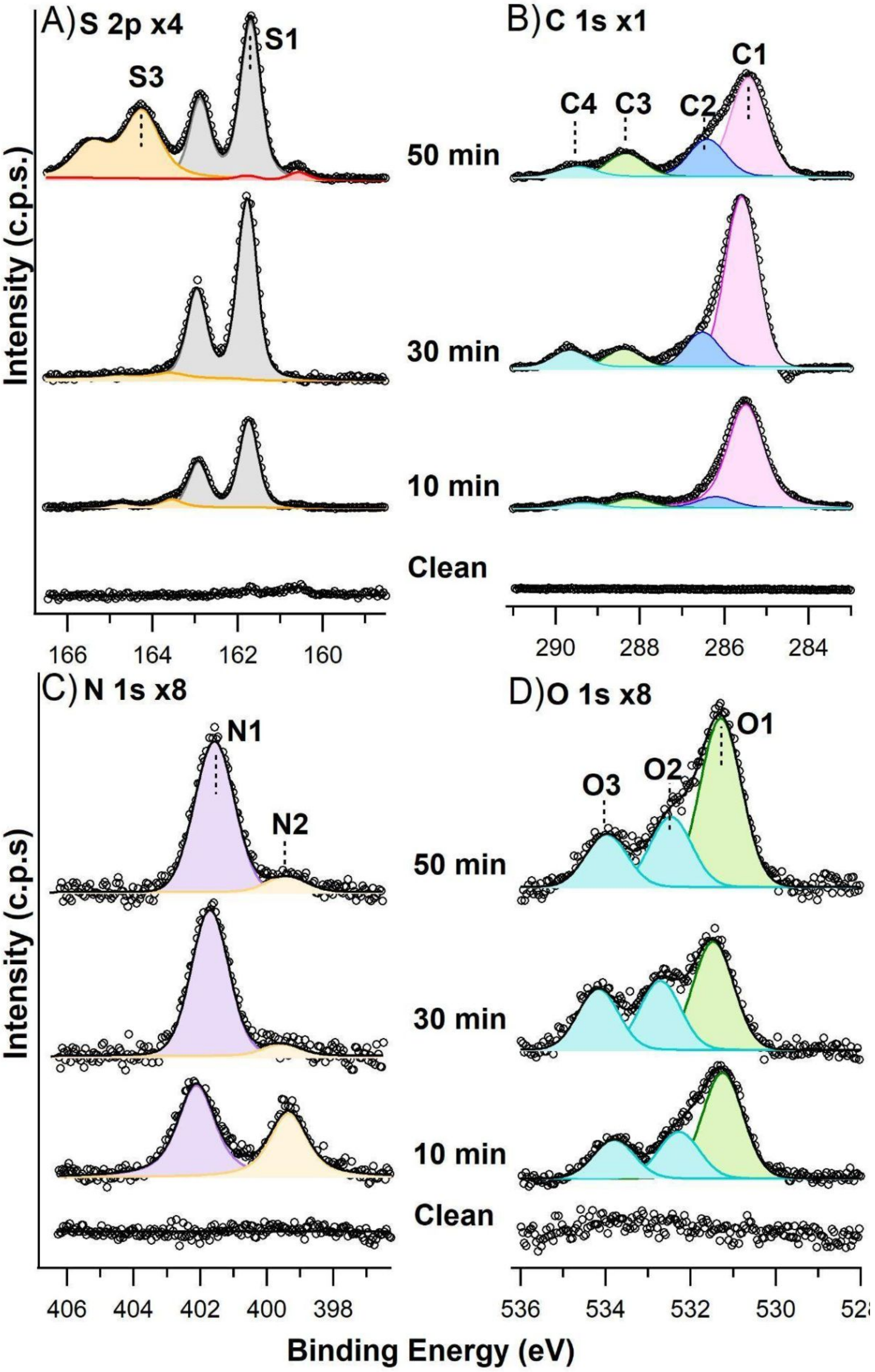

Figure S1. HR-XPS spectra of (L)-Cysteine/Ag(110) system recorded after incremental Cys doses on the surface at RT. Panels A)-D) show the S 2p, C 1s, N 1s and O 1s regions, respectively. Note the magnification factor marked for each region.

Figure S1 shows the evolution of the HR-XPS spectra during (L)-Cysteine uptake on Ag(110) at RT. The uptake curves (total area of the O 1s, N 1s and S 2p envelopes vs exposure time) and the relative concentration of these elements at the surface are reported in Figure S2.

We remark that:

i) The total intensity of the O 1s, N 1s and S 2p envelopes, that is proportional to the amount of the corresponding element at the surface, increases almost linearly with exposure time, as evident from inspection of the spectra in figure S1 and from the associated analysis of Figure S2A. The relative amount of the three elements (Figure S2B) is compatible with the 2:1:1 ratio expected from the Cys stoichiometry. Small deviations from the nominal value as well as small differences in the slope of the uptake curves may be related to the particular molecular conformation. Therefore, the Cys units adsorb non-dissociatively over the whole coverage range explored.

ii) The assignment of the different components present in each region reflects the one reported in the main text, with the exceptions remarked in the following discussion.

iii) The C 1s spectrum of the clean surface presents a small residual contamination, that was subtracted from all C 1s spectra for the analysis. Despite that, the C1 line at 285.5 eV is always significantly higher than the others, at variance with what is observed in the corresponding spectrum in the main text (see bottom trace in Figure 3). This is indicative that C contamination occurs during the uptake and that its intensity is superimposed to the lowest E_b component of the cysteine molecules. We mention that this is the first sublimation experiment performed during the beamtime and therefore traces of C contamination during evaporation can be still present. Indeed, despite the evaporator was outgassed at the best of our possibilities, some residual contamination can still arise from the crucible or from the Ta filament used for heating, that is wrapped around the crucible and exposed to vacuum. The following preparations, as the one reported in Figure 3 of the manuscript, show the expected ratio among the C 1s components and a much lower excess of C with respect to the other Cys elemental constituents.

Nonetheless, the uptake of Figure S1 can provide additional information from the O1s, N1s and S 2p regions and it is therefore worth showing it as supporting material.

iv) The S 2p region (panel A) is characterized by the usual doublet at E_b(S $2p_{1/2}$) = 161.7 eV, present already after the first dose and corresponding to the thiolate bound to Ag [1], and by an additional doublet at E_b(S $2p_{3/2}$) = 164.2 eV (S3). Since the latter species appears only after the largest exposure, it is indicative that a different adsorption configuration sets in to accommodate more molecules. Its binding energy is suitable for protonated sulfur atoms [1] but also for S atoms in a -S-S- bridge in cystine [2]. Therefore, the S3 doublet could be ascribed either to the initial nucleation of a second, more weakly bound Cys layer or to the formation of some cysteine dimers.

The presence of some bilayer cannot be ruled out but multilayer formation in significant amount should cause changes in the C 1s spectrum larger than those observed [1,3]. On the other hand, bulk cystine has a binding energy of 163.4 eV for S 2p while the C 1s spectrum presents an envelope with different components between 284.6 and 287.9 eV. Since the S atoms in the -S-S- bridge of cystine are poorly interacting with the surface, their S 2p binding energy should be little affected by adsorption, that makes the observed S3 signal compatible with this assignment. We also observe that the O 1s of cystine has a main component around 530.5 eV, that is coherent with the larger relative intensity of the O1 component in the 50 minute evaporation spectrum with respect to the shorter exposures. Additional experiments would be required to determine the relative amount of Cys bilayer and/or of Cystine at the surface above a critical coverage, but this is out of the scope of the present study, that is focused on the self-assembly of Cys monolayers.

v) The N 1s region is characterized by a main peak centered around $E_b \sim 402.0$ and slightly downshifting with increasing exposure. As discussed in the main text, it corresponds to protonated amino groups (NH_3^+) and indicates that Cys molecules adsorb on Ag(110) at RT mainly in the zwitterionic form [1] already in the initial stages of the deposition process. The presence of an additional peak at $E_b = 399.3$ eV may indeed correspond to the population of sites stabilizing adsorption in the anionic form [3] but, in our case, a photon-induced effect converting the zwitterion into neutral NH_2 groups is likely to contribute to a large fraction of its intensity. This conclusion is achieved by comparing the N 1s spectra reported in Figure S3, recorded after the same 10 minute exposure of Figure S1. The red spectrum was recorded after prolonged exposure of the organic layer to the photon beam, while the black one was acquired shortly after moving to an unexposed sample area. As evident, the intensity of the 399.3 eV peak is enhanced after prolonged irradiation. The increased exposure to X-rays also justifies why, in the uptake experiment of Figure S1, the N2 peak is larger for the shortest exposure.

To minimize this unwanted effect, all the subsequent spectra of Figure S1 and of Figure 3 were recorded on a virgin, not previously illuminated area.

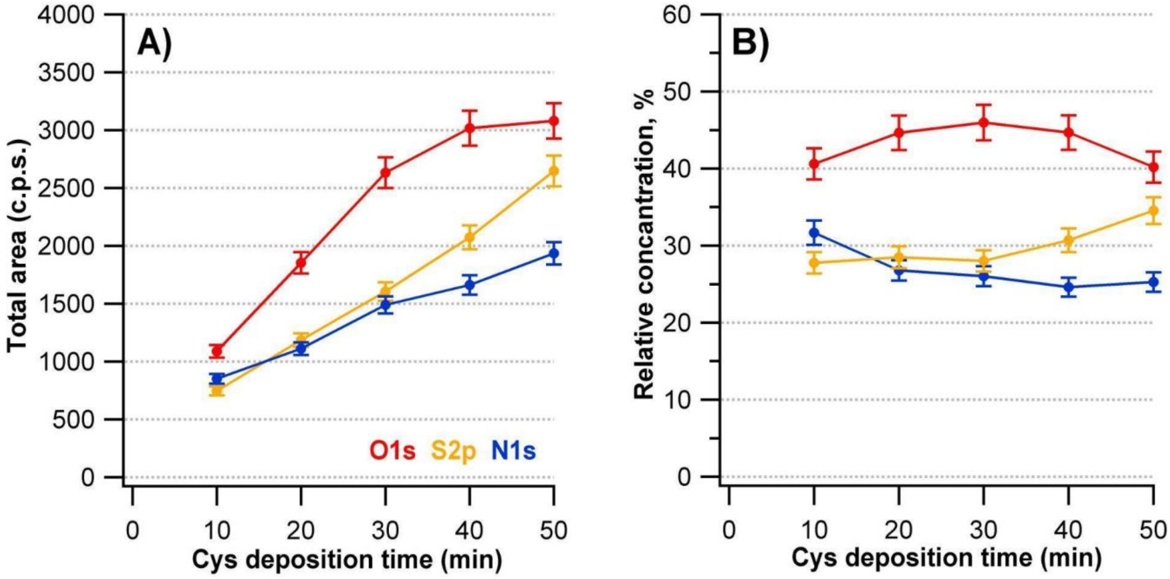

Figure S2. Total area (A) and the relative concentration (B) of O, N and S atoms at the surface versus Cys deposition time, as estimated from the spectra of Figure S1. All data points are affected by an estimated error of ±5%. The areas and relative concentrations of all elements are scaled on the corresponding photoionization cross sections for direct comparison [4].

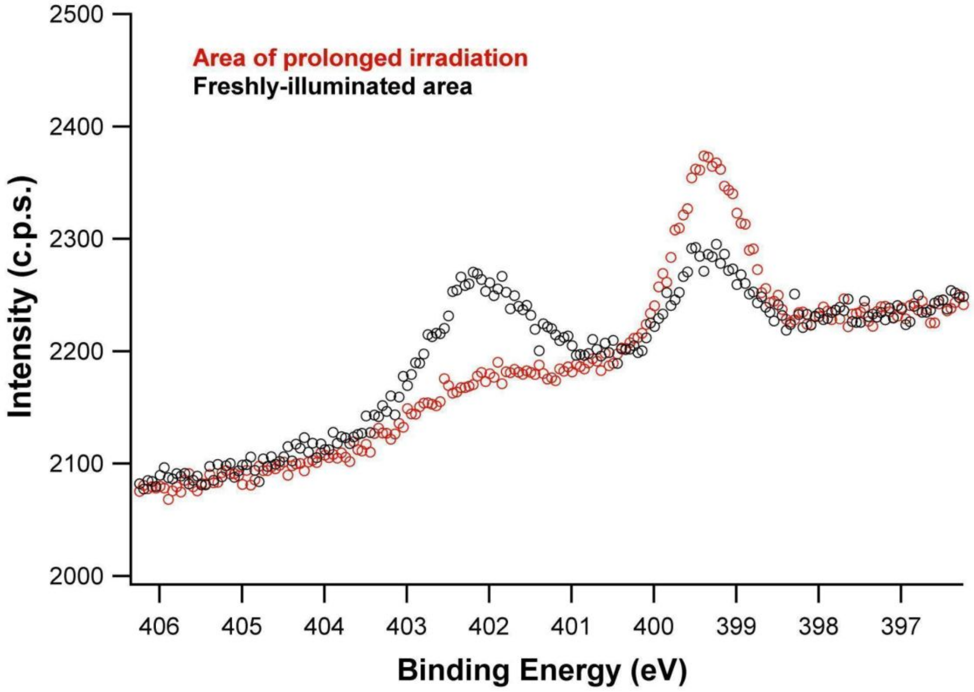

Figure S3. HR-XPS spectra of the N 1s region recorded on the Cys/Ag(110) after 10 minutes deposition at RT. Comparison of traces recorded on an area of prolonged irradiation and on a freshly-illuminated area.

1. Fischer, S.; Papageorgiou, A.; Marschall, M.; Reichert, J.; Diller, K.; Klappenberger, F.; Allegretti, F.; Nefedov, A.; Wöll, C.; Barth, J. l-Cysteine on Ag(111): A Combined STM and X-ray Spectroscopy Study of Anchorage and Deprotonation. *J. Phys. Chem. C* **2012**, *116*, 20356-20362.

2. Salles, R. C. M.; Coutinho, L. H.; Da Veiga, A. G.; Sant'Anna, M. M.; De Souza, G. G. B. Surface Damage in Cystine, an Amino Acid Dimer, Induced by KeV Ions. *J. Chem. Phys.* **2018**, *148,* 045107.

3. Gonella, G.; Terreni, S.; Cvetko, D.; Cossaro, A.; Mattera, L.; Cavalleri, O.; Rolandi, R.; Morgante, A.; Floreano, L.; Canepa, M. Ultrahigh Vacuum Deposition of L-Cysteine on Au(110) Studied by High-Resolution X-Ray Photoemission: From Early Stages of Adsorption to Molecular Organization. *J. Phys. Chem. B* **2005**, *109* (38), 18003–18009.

4. Atomic Calculation of Photoionization Cross-Sections and Asymmetry Parameters, available at VUO / Elettra: https://vuo.elettra.eu/services/elements/WebElements.html
